# Spanish real-world experience with fingolimod in relapsing-remitting multiple sclerosis patients: MS NEXT study

**DOI:** 10.1371/journal.pone.0230846

**Published:** 2020-04-02

**Authors:** Francisco Barrero, Javier Mallada-Frechin, María Luisa Martínez-Ginés, María Eugenia Marzo, Virginia Meca-Lallana, Guillermo Izquierdo, José Ramón Ara, Celia Oreja-Guevara, José Meca-Lallana, Lucía Forero, Irene Sánchez-Vera, María José Moreno, in representation of the MS NEXT study investigators

**Affiliations:** 1 Neurology Department, Hospital Uniersitario San Cecilio de Granada, Granada, Spain; 2 Neurology Department, Hospital General Universitario de Elda, Alicante, Spain; 3 Neurology Department, Hospital General Universitario Gregorio Marañón, Madrid, Spain; 4 Neurology Department, Hospital San Pedro, Logroño, Spain; 5 Demyelinating Disorders Unit, Hospital Universitario de La Princesa, Madrid, Spain; 6 Neurology Department, Hospital Universitario Virgen Macarena, Sevilla, Spain; 7 Neurology Department, Hospital Universitario Miguel Servet, Zaragoza, Spain; 8 Neurology Department, Hospital Universitario Clínico San Carlos, Madrid, Spain; 9 Hospital Virgen de la Arrixaca (IMIB-Arrixaca), Murcia, Spain; 10 Neurology Department, Hospital Universitario Puerta del Mar, Cádiz, Spain; 11 Novartis Farmacéutica S.A., Barcelona, Spain; University of Ioannina School of Medicine, GREECE

## Abstract

**Purpose:**

The objective of this study was to characterize the demographic and clinical profile of RRMS patients receiving fingolimod in Spain, and to evaluate drug effectiveness and safety in clinical practice.

**Methods:**

This observational, retrospective, multicentre, nationwide study was performed at 56 Spanish hospitals and involved 804 RRMS patients who received oral fingolimod (0.5 mg) since November 2011, with a minimum follow-up of 12 months.

**Results:**

The mean annualized relapse rate (ARR) in the year before fingolimod was 1.08 and the median EDSS was 3; patients were exposed to fingolimod for 2.2 years as average; regarding magnetic resonance imaging (MRI) activity, more than half of the patients had >20 lesions at baseline. Patients were previously treated with first-line injectable DMTs (60.3%), or natalizumab (31.3%), and 8.3% were naïve patients. Overall, the ARR significantly decreased to 0.28, 0.22 and 0.17 (74.1%, 79.7% and 83.5% of relative reduction, respectively) after 12, 24 and 36 months of treatment, *P*<0.001. The ARR of patients who switched from natalizumab to fingolimod was stable over the study. Most of the patients (88.7%) were free from confirmed disability and MRI activity (67.3%) after 24 months. The persistence after 12 months on fingolimod was 93.9%.

**Conclusions:**

The subgroups of patients analysed showed differential baseline demographic and clinical characteristics. The analysis of patients who received fingolimod in routine clinical practice confirmed adequate efficacy and safety, even for long-term treatment. The present data also confirmed the positive benefit/risk balance with fingolimod in real-world clinical practice setting.

## Introduction

Multiple sclerosis (MS) is the most common chronic, immune‑mediated, and neurodegenerative disease in young adults.[1,2] Fingolimod (Gilenya®, Novartis Pharma AG), a sphingosine 1-phosphate receptor modulator, was the first oral drug approved in Spain for treating remitting-relapsing multiple sclerosis (RRMS) patients.[3] Fingolimod acts as a functional antagonist of the sphingosine-1-phosphate type 1 receptor, inducing receptor internalization and rendering T and B cells insensitive to the egress signals from secondary lymphoid tissues. The resulting lymphocyte redistribution reduces recirculation of auto-aggressive cells to the central nervous system.[4] Due to the lipophilic nature of the drug, fingolimod crosses the blood–brain barrier, and is phosphorylated within the central nervous system.[5] The efficacy of fingolimod was proved in 3 phase III randomized clinical trials: FREEDOMS, FREEDOMS II, and TRANSFORMS,[6–8] showing higher efficacy in reducing annual relapse rate (ARR), disability worsening, and magnetic resonance imaging (MRI) activity compared to placebo and over interferon (IFN) beta 1-a. Additionally, fingolimod reduced brain volume loss (BVL), suggesting its potential dual action over the disease inflammation and neurodegeneration.[9] Adherence to disease-modifying therapies (DMTs) have been associated to decreased risk of relapses and to the improved health-related quality-of-life.[10] It has been reported that patients on fingolimod had higher treatment compliance than patients on injectable DMT (iDMT).[11] To date, there are limited multicentre studies conducted in large cohorts of patients within the real-world setting. The objective of the present study was to characterize the demographic and clinical profile of RRMS patients receiving fingolimod, and to evaluate the drug effectiveness and safety after at least 12 months of follow-up in the Spanish clinical practice setting.

## Materials and methods

### Study design

This observational, retrospective, multicentre, nationwide study involved patients with RRMS who received treatment with oral fingolimod (Gilenya^®^ 0.5 mg) between November 2011 and December 2015. The study was performed in 56 hospitals, in Spain.

Inclusion criteria were: women and men older than 18; with diagnosis of RRMS according to McDonald criteria 2010;[12] having initiated treatment with fingolimod, with available follow-up data from a period of, at least, 12 months, and with sufficient data in the clinical history. Patients were included according to the following criteria: 1) patients with high disease activity despite a complete course of treatment with, at least, one DMT, or “non-responder” patients defined as patients who experienced ≥ 1 relapse one year before fingolimod initiation (while receiving a previous DMT), and that had ≥ 9 T2 lesions in the baseline brain MRI, or ≥1 gadolinium-enhancing MRI lesion; or as patients with an equal or greater ARR compared with the previous year; 2) naïve patients with rapid evolving MS (defined by ≥2 relapses in a year, and ≥1 gadolinium-enhancing T1 lesions on the baseline brain MRI- or a significant increase in the T2 lesion load, compared with a previous recent MRI); and 3) patients who initiated fingolimod after withdrawal of natalizumab (NTZ). Exclusion criteria were: patients who had received fingolimod, as part of a clinical trial, before the inclusion in the study; or with clinical manifestation of MS other than RRMS. Treatment with fingolimod could be temporarily or permanently discontinued during the 12 months of follow-up.

Recruitment started in November 2014 and ended in December 2015. Procedures were in concordance with the guidelines established in the Declaration of Helsinki, and performed following Good Clinical Practice guidelines.

The Clinical Research Ethics Committee of the Hospital Clinic of Barcelona was the reference ethics committee which approved the study in September 2014 (EPA Reg. HCB/2014/0849). In the other study sites, the study was approved by their respective ethics committees or by the hospital management, depending on the internal procedures of each site. Given the retrospective nature of this study design, informed consent was not obtained and not requested by ethics committees.

### Groups and variables

Patients were stratified in 3 subgroups according to previous treatments: those previously treated with iDMT(s) as first-line treatment(s) (including subcutaneous or intramuscular IFN beta-1a, subcutaneous IFN beta-1b and subcutaneous glatiramer acetate (GA); named Post-iDMTs), patients previously treated with NTZ (Post-NTZ), or patients who did not receive any DMT in the previous year (Naïve). Naïve patients included patients who did not receive any previous treatment (aggressive RRMS) and those who received a previous DMT (at least 24 months prior to fingolimod initiation).

The level of disability was determined by using the Expanded Disability Status Scale (EDSS).[13] Time to 3-month CD (confirmed disability) was defined as an increase of ≥1 point in the EDSS score when baseline EDSS ≥1, or an increase of ≥1.5 points, when baseline EDSS = 0, confirmed after 3 months.

Sustained improvement was defined as the reduction in ≥1 point in the EDSS score, and maintained for at least 3 months. Adverse events (AEs) were collected according to fingolimod risk management plan.

### Sample size and statistical analysis

Sample size calculation was performed with the resource “Java applets for power and sample size”.[14] A sample size of 800–900 patients provided sufficient statistical power to measure the treatment effectiveness with an error equal to 0.02 (for the 95% CI) and detect an AE occurring with a frequency ≥ 0.3% (with a ±0.27 error for a 95% CI).

The 3-month CD and time to relapses were analysed using the Kaplan-Meier methodology; the differences between groups were compared using the log-rank test. Demographic and clinical characteristics of patients were compared among groups by the non-parametric Kruskal-Wallis or Mann-Whitney-Wilcoxon tests, when appropriate.

The baseline influence of sex, age, number of T2 and T1 gadolinium-enhancing (Gd+) lesions, and EDSS on the ARR reduction was evaluated using the analysis of covariance (ANCOVA). The ANCOVA model included the ARR in previous year as covariable, and the following factors: time, previous treatment, and its interaction. Differences among groups regarding sustained improvement, stability or disease worsening were analysed using the Fisher’s Exact test. Statistical significance was established with *P* < 0.05. All statistical procedures were performed by using SAS v9.4 software (SAS Institute Inc., Cary, NC).

## Results

### Baseline characteristics of patients

A total of 872 patients were initially recruited and 804 were considered evaluable. Sixty-eight patients (7.8%) were excluded for: not having the minimum clinical information in the medical records (n = 47); or having received a previous treatment different to IFN beta, GA, or NTZ (n = 21).

The majority of patients were female (68.5%), with a median age of 41.0 years (SD 9.1). Patients initiated treatment with fingolimod 8.9 years (SD 6.0) after RRMS diagnosis and were on fingolimod 2.2 years (SD 0.8) (Table 1).

**Table 1:** Baseline demographic and clinical characteristics for the total population and for patient subgroups regarding previous treatment.

	Post- iDMTs(N = 485, 60.3%)	Post-NTZ(N = 252, 31.3%)	Naïve(N = 67, 8.3%)	Total(N = 804)	P-value (inter-groups)
Age, mean years (SD) Range (min-max)	41.2 (9.1)	41.4 (8.8)	38.6 (9.7)	41.0 (9.1)	0.079
	20–69	20–63	18–64	18–69	
Sex female, n (%)	347 (71.6)	162 (64.3)	42 (62.7)	551 (68.5)	0.074
Time from RRMS diagnosis to fingolimod initiation, mean years (SD)	8.6 (5.9)	10.4 (5.5)	4.6 (6.5)	8.9 (6.0)	<0.001
Duration of treatment with fingolimod, mean years (SD)	2.1(0.8)	2.2 (0.9)	2.1 (0.9)	2.2(0.8)	N.A.
Annualized relapse rate at previous year, mean (SD)	1.28 (0.87)	0.33 (0.75)	2.47 (1.82)	1.08 (1.13)	<0.001
Number of relapses at 2 previous years, mean (SD)	1.8 (1.1)	0.4 (0.9)	2.0 (1.0)	1.4 (1.2)	<0.001
EDSS, mean (SD) Median (IQR)	2.9 (1.5)	3.5 (1.8)	2.7 (1.8)	3.1 (1.7)	<0.001
	2.5 (2.0–4.0)	3.5 (2.0–4.5)	2.5 (1.5–3.5)	3.0 (2.0–4.0)	
Number of gadolinium-enhanced T1 lesions, mean (SD)*	1.3 (2.5)	0.3 (1.2)	1.7 (2.6)	1.2 (2.4)	<0.001
Patients with T2 lesions, n (%) **					
<9 lesions	24 (8.1)	4 (7.4)	7 (17.5)	35 (8.9)	0.160
9–20 lesions	113 (37.9)	28 (51.9)	13 (32.5)	154 (39.3)	0.101
>20 lesions	161 (54.0)	22 (40.7)	20 (50.0)	203 (51.8)	0.193

iDMTs, injectable disease-modifying therapies; NTZ, natalizumab; RRMS, relapsing-remitting multiple sclerosis; N.A., not available; EDSS, expanded disability status scale; IQR, interquartile range

* Calculated over 307, 57, and 40 patients with available information from Post- iDMTs, Post-NTZ, and Naïve groups, respectively.

** Calculated over 298, 54, and 40 patients with available information from Post- iDMTs, Post-NTZ, and Naïve groups, respectively.

The number of previous DMTs in the Post-NTZ (2.6, SD 0.9) was significantly higher than in the Post-iDMTs patients (1.7, SD 0.9). The main reason for initiating treatment with fingolimod was the lack of effectiveness of the previous treatment (58.3% of patients), followed by the increased risk of progressive multifocal leukoencephalopathy (PML) with NTZ (28.9%). At study initiation, the median EDSS was 3.0 (IQR, 2.0–4.0; Table 1). The mean ARR one year before fingolimod initiation was 1.08 (SD 1.13); in the Naïve group the ARR (2.47, SD 1.82) was significantly higher than in both the Post-iDMTs (1.28, SD 0.87) and Post-NTZ groups (0.33, SD 0.75; *P*<0.001; Table 1). The mean number of gadolinium-enhancing T1 lesions was 1.2 (SD 2.4) and half of the patients (51.8%) showed > 20 T2 lesions at baseline (Table 1). The mean number of gadolinium-enhancing T1 lesions in the Post-iDMTs (1.3, SD 2.5) and the Naïve groups (1.7, SD 2.6) was significantly higher than the Post-NTZ group (0.3, SD 1.2; *P*<0.001) (Table 1). Approximately half of the patients in the Post-NTZ group (51.9%) showed between 9 and 20 T2 lesions at baseline, whereas most of the patients in the Post-iDMTs (54%) and Naïve groups (50%) showed more than 20 lesions.

### Evaluation of effectiveness

#### Relapses

**Fig 1:**
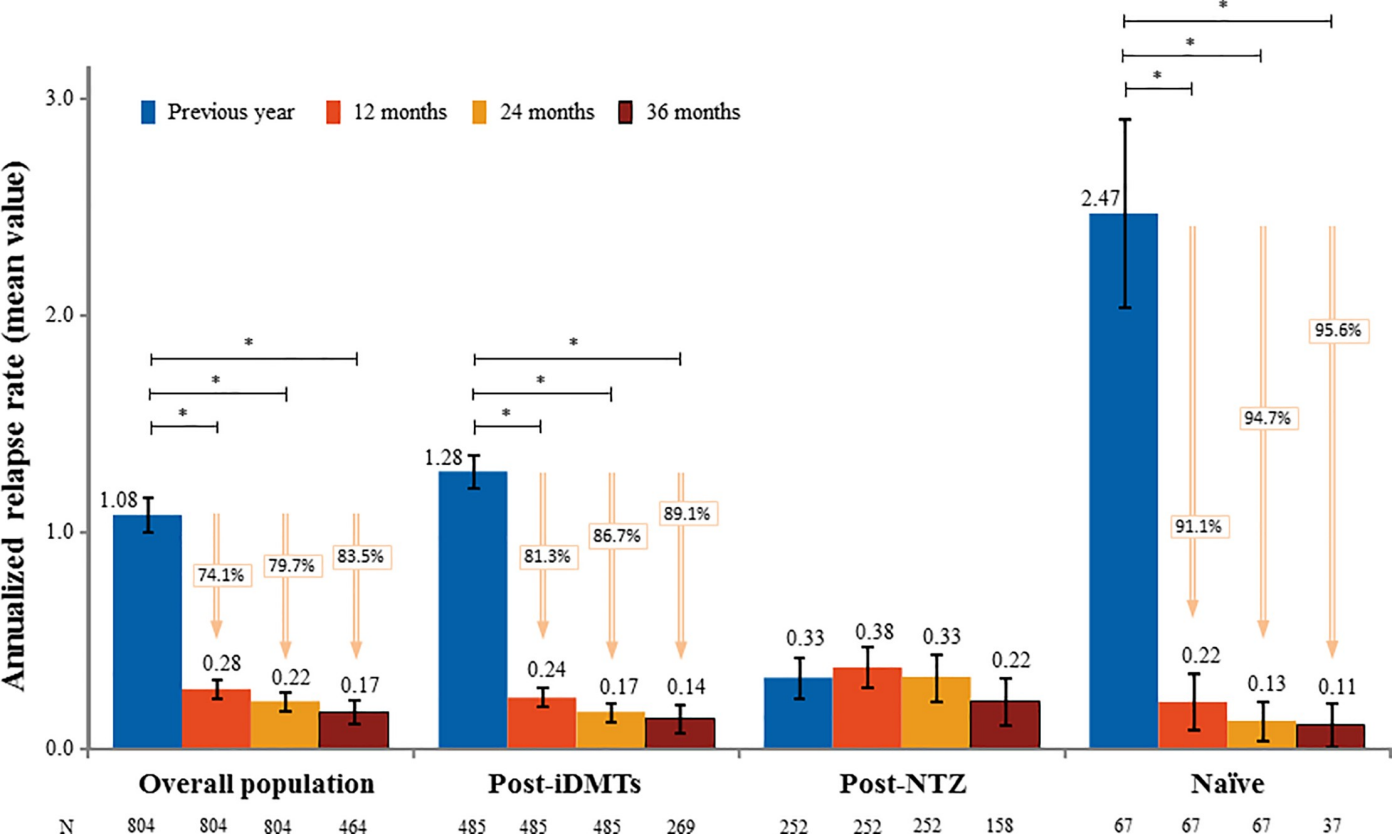
Annualized relapse rate (ARR) one year before initiation and after 12, 24 and 36 months of treatment with fingolimod. 95% confidence intervals are shown (black lines). * p-value < 0.05; iDMTs, injectable disease-modifying therapies; NTZ, natalizumab.

The ARR significantly decreased from 1.08 (SD 1.13), one year before treatment initiation, to 0.28 (SD 0.61; -74.1%; *P*<0.001), 0.22 (SD 0.64; -79.7%; *P*<0.001), and 0.17 (SD 0.60; -83.5%; *P*<0.001) after 12, 24 and 36 months of treatment with fingolimod, respectively (Fig 1 and S1 Table). Patients with a baseline EDSS≤3 showed a reduction in the ARR after 24 months significantly higher than those with a baseline EDSS>3 (-0.10 [SD = 0.05], p = 0.0212). Regarding patient subgroups, the ARR significantly decreased for Post-iDMTs and Naïve patients after 12 (0.24, SD 0.50, -81.3%; and 0.22, SD 0.60, -91.1%; respectively; *P*<0.001), 24 (0.17, SD 0.48; -86.7%; and 0.13, SD 0.40, -94.7%; *P*<0.001), and 36 months of treatment (0.14, SD 0.50, -89.1%; and 0.11, SD 0.40, -95.6%; *P*<0.001). The ARR in the Post-NTZ group remained stable during the 36 months of treatment (0.38, SD 0.75 after 12; 0.33, SD 0.89 after 24; and 0.22, SD 0.70 after 36 months; p<0.05 in all cases; Fig 1). A total of 534 patients (66.4%) had no relapse during the treatment with fingolimod. The first relapse occurred 26.6 months (SD 0.5) after fingolimod initiation in 33.6% of the patients. The median time elapsed between the treatment initiation and the first relapse in the Post-NTZ group was significantly lower than in the Post-iDMTs and Naïve groups (*P* = 0.024; S1 Table).

#### Disability

In all the groups analysed (post-iDMTs, post-NTZ, naïve patients), the majority of patients showed a sustained stability at 12 months (post-iDMTs 72.3%; post-NTZ 82.9%; naïve 62.5%) and at 24 months (post-iDMTs 65.1%; post-NTZ 73.7%; naïve 64.7%) in disability.

**Fig 2:**
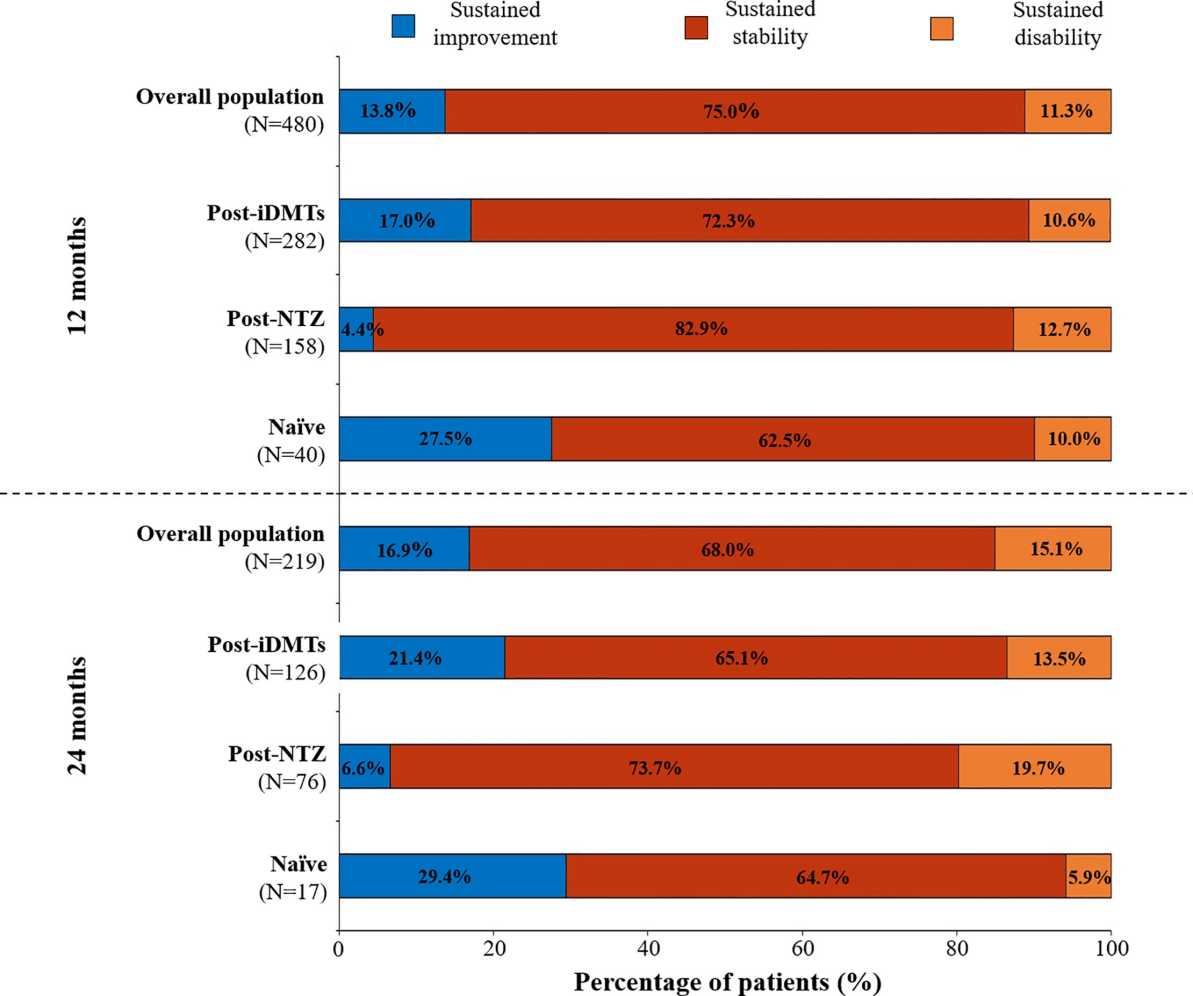
Change in the Expanded Disability Status Scale (EDSS) between one year before treatment initiation and 12 and 24 months of treatment with fingolimod. iDMTs, injectable disease-modifying therapies; NTZ, natalizumab.

Although the percentage of patients with disability was similar between groups (11.3% of total patients), the percentage of patients with sustained improvement after 12 months in the Post-iDMTs (17.0%) and Naïve groups (27.5%) was numerically higher than in Post-NTZ group (4.4%) (Fig 2).

#### Radiological activity

The number of T1 Gd+ lesions significantly (P<0.001) decreased from 1.2 to 0.4 (SD 1.1) after 12 months, and to 0.1 (SD 0.3) after 24 months in the total population and also in all the patient subgroups (post-iDMTs, post-NTZ and naïve) analysed (S1 Table). A 74.9% of patients were free from new/enlarged T2 lesions after 12 and 24 months on fingolimod and 69.7% after 36 months (S1 Table). Data obtained after 36 months of treatment, should be interpreted cautiously due to the reduced number of MRI data available.

#### Disease activity

**Fig 3:**
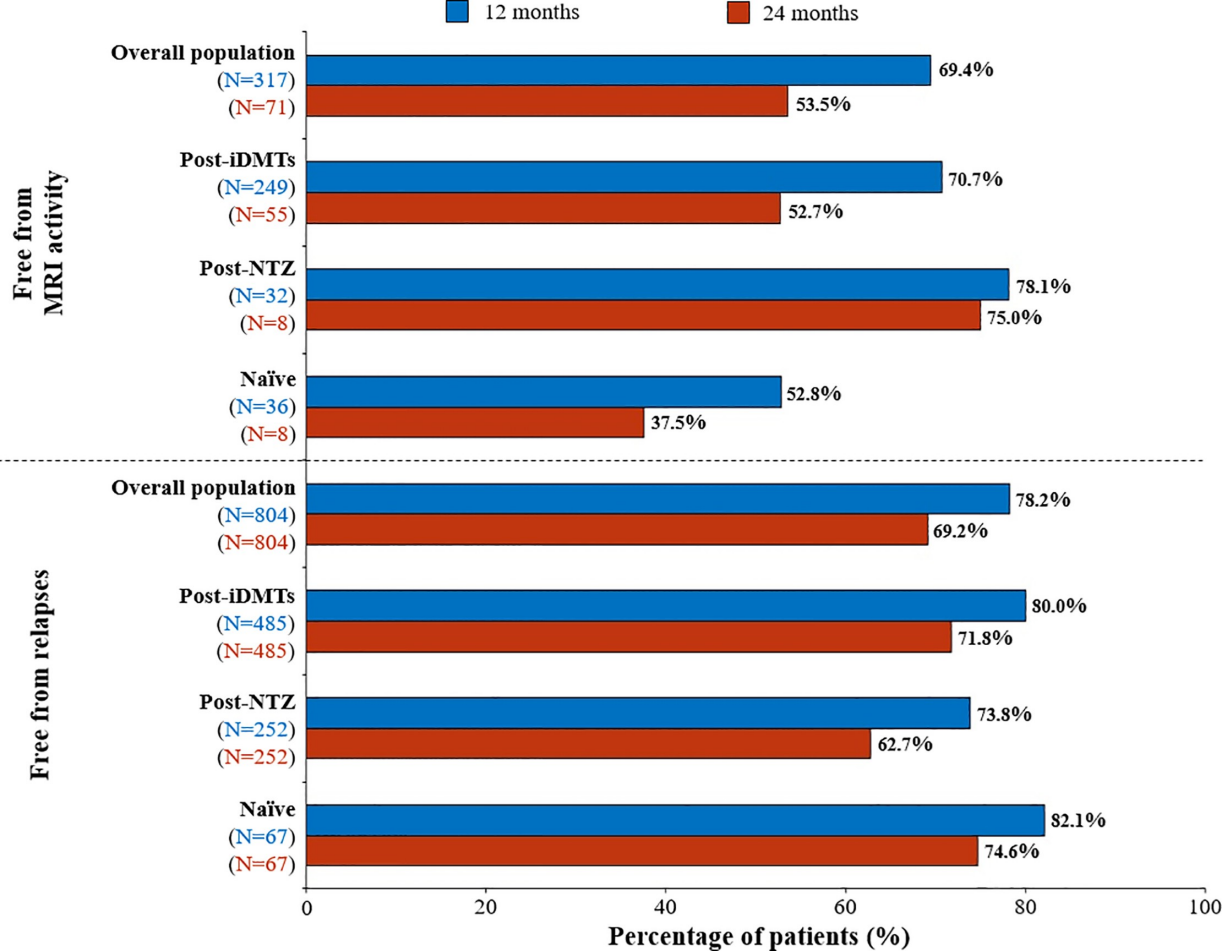
MRI and relapse-free patients after 12 and 24 months of treatment with fingolimod. iDMTs, injectable disease-modifying therapies; NTZ, natalizumab.

The percentage of relapse-free patients after 12 and 24 months of treatment with fingolimod was 78.2% and 69.2%, respectively. After 12 and 24 months of treatment, the percentage of relapse-free patients was for Post-iDMTs 80.0% and 71.8%, for Naïve 82.1% and 74.6% and for Post-NTZ group 73.8% and 62.7% (Fig 3 and S1 Table).

The percentage of patients free from clinical activity after 12 and 24 months were 77.7% and 68.3%, in the Post-iDMTs 77.6% and 68.6%in the Naïve group, and 69.4% and 56.8%in the Post-NTZ group, respectively. Finally, the percentage of disease-free patients was 53.8% and 38.0%, after 12 and 24 months of fingolimod treatment, respectively (S1 Fig and S1 Table). The percentage of patients free from MRI was 69.4% after 12 months of treatment, and 53.5% after 24 (Fig 3). The majority of the patients (88.7%) were free from CD during the follow-up (S1 Table). The mean time to 3-month CD was 32.5 months (SD, 0.3 months) after treatment initiation. The percentage of patients free from disease was 53.8% and 38.0%, after 12 and 24 months respectively (S1 Fig and S1 Table). After 12 and 24 months of treatment with fingolimod, the majority of Post-iDMTs (99.8% and 90.1%), Naïve (98.4% and 93.1%) and Post-NTZ patients (97.5% and 85.7%) were CD-free respectively. No information after 36 months of treatment was provided due to the low number of patients.

#### Safety profile and treatment persistency

Most of the patients (98.3%) did not suffer any AE following the first dose of fingolimod (Table 2); a 1.7% of the patients reported at least one adverse event, such as atrioventricular block (0.9%) or bradycardia (0.4%). After fingolimod initiation 20% of patients reported AEs, mostly infections (7%), liver enzymes increase (4.6%), and lymphopenia < 200 cells/μl (2.5%). The persistence with fingolimod after 12 months was 93.9% and was similar among groups (90.7% in Post-iDMTs, 86.9% in Post-NTZ, and 91.0% in Naïve). An 8.6% (n = 69) of the patients discontinued fingolimod during the first 12 months of treatment: 5.7% (n = 46) permanently, and 3.9% (n = 31) temporarily. Main reason for temporary discontinuation during the study period were: AEs (29 patients, 3.6%), patient decision (9 patients, 1.1%), and pregnancy (4 patients, 0.5%); whereas for permanent discontinuation were AEs (22 patients, 2.7%), and lack of effectiveness (19 patients, 2.4%).

**Table 2:** Adverse events reported during the treatment with fingolimod for the total population and for patient subgroups regarding previous treatment.

	Post- iDMTs(N = 485)	Post-NTZ(N = 252)	Naïve(N = 67)	Total(N = 804)
**After the first dose of fingolimod**, n (%)				
Patients with any adverse event *	9 (1.9)	3 (1.2)	2 (3.0)	14 (1.7)
Bradycardia **	1 (0.2)	1 (0.4)	1 (1.5)	3 (0.4)
Atrioventricular block	6 (1.2)	1 (0.4)	0 (0.0)	7 (0.9)
Others	2 (0.4)	1 (0.4)	1 (1.5)	4 (0.5)
Asthenia / Fatigue	1 (0.2)	1 (0.4)	0 (0.0)	2 (0.2)
Nausea	1 (0.2)	0 (0.0)	0 (0.0)	1 (0.1)
Dizziness	0 (0.0)	0 (0.0)	1 (1.5)	1 (0.1)
**After subsequent doses of fingolimod,** n (%)				
Patients with any adverse event *	96 (19.8)	55 (21.8)	10 (14.9)	161 (20.0)
Infections	31 (6.4)	25 (9.9)	0 (0.0)	56 (7.0)
Liver enzymes increase	24 (5.0)	10 (4.0)	3 (4.5)	37 (4.6)
Lymphopenia (< 200 cells/μl)	13 (2.7)	5 (2.0)	2 (3.0)	20 (2.5)
Macular oedema	1 (0.2)	2 (0.8)	0 (0.0)	3 (0.4)
Pregnancy	1 (0.2)	1 (0.4)	0 (0.0)	2 (0.3)
Hypersensitivity	2 (0.4)	0 (0.0)	0 (0.0)	2 (0.3)
Symptomatic bradycardia	0 (0.0)	0 (0.0)	0 (0.0)	0 (0.0)
Atrioventricular block	0 (0.0)	0 (0.0)	0 (0.0)	0 (0.0)
Others (no specified)	52 (10.7)	37 (14.7)	9 (13.4)	98 (12.2)

iDMTs, injectable disease-modifying therapies; NTZ, natalizumab.

* Adverse events occurred the same day or the following day to the first dose of fingolimod (initiation or re-initiation of the treatment).

** Two bradycardia were symptomatic, and one was prolonged asymptomatic.

## Discussion

To date there are limited studies aimed to describe the demographic and clinical profile of patients receiving fingolimod in clinical practice, and to evaluate its effectiveness and safety in large cohorts of patients.[10,15–21] Moreover, methodologies of these studies are heterogeneous: performed in single Health Centre;[19–21] using diverse data sources (administrative claims, registries, medical records);[10,15–25] with scarce and variable number of patients (most of them < 250);[19–21] with different MS types (RRMS, progressive MS);[17–18] and/or diverse follow-up periods.[16,19] The present MS NEXT represents the most extensive clinical practise study nationally performed so far with fingolimod.

### Overall population

According to our results, the patient candidate to initiate treatment with fingolimod was characterized by a high disease activity, i.e. ARR, EDSS, disability, and high MRI activity. This patient profile was more severe than found in clinical trials such as FREEDOMS, FREEDOMS II, and TRANSFORMS studies.[6–8] Besides this, fingolimod has demonstrated to be effective in real-word settings by reducing ARR and MRI lesions, and stabilising EDSS. In the first year of treatment, fingolimod produced a marked reduction in the ARR, which remained stable for the 3 years of follow-up, a stabilization of EDSS, and a decrease in MRI lesions (T1 Gd+ and T2 lesions). The MRI results (from the second year on) should be cautiously interpreted due to the low number of available samples. The limitation of long-term MRI data was not related to the withdrawal of the treatment. Indeed, diverse studies have demonstrated higher rates of treatment adherence and persistence with fingolimod.[11] However, in clinical practice, MRI evaluation of stable patients may not be performed annually and T1 Gd+ lesions may not be systematically analysed in every centre. Furthermore, not all the patients received fingolimod for 3 years (some of them initiated the treatment later than others).

### Naïve population

In our study, naïve patients initiating treatment with fingolimod were younger and with a shorter time of disease evolution (from diagnosis) than overall population and other subgroups. Nevertheless, the naïve patients treated with fingolimod were the highly active, with higher inflammatory activity, such as relapses and MRI lesions; showing that the choice is still for later lines of treatments, although it has been suggested that earlier treatment is better.[26]

Regarding ARR reduction, naïve patients showed better outcomes than other subgroups probably due to the higher baseline ARR in this subgroup of patients. Despite this, ARR remained low during the treatment with fingolimod (from first to third year). Similarly, most of naïve patients showed improvement in EDSS, or remained stable, through the study period. Again, careful conclusions should be made with EDSS and MRI findings from second year due to the low number of available samples. The limitation of this information may have occurred for several reasons: not all the patients received fingolimod for 3 years (they could initiate later the treatment); it was necessary a 3-month confirmed EDSS; and EDSS or MRI evaluation of stable patients may not be routinely performed in clinical practice. The best outcomes obtained in these naïve patients corroborate that early treatment with fingolimod is effective for modifying the course of the disease in the clinical practice settings, and provides long-term benefits for the patient.

### Post-NTZ population

According to our results, post-NTZ patients had higher time of disease evolution and EDSS at baseline than other subgroups. Moreover, at this time they had a lower ARR and MRI activity presumably because they had already received a high-efficacy drug (most of them received 2 or more previous treatments). The lack of effectiveness of the previous treatment and the increased risk of progressive multifocal leukoencephalopathy were the main reasons for switching to fingolimod. The washout period between NTZ withdrawal and the initiation of fingolimod was heterogeneous (mean 4.52 months, SD 4.2) and was excluded from the analysis of all variables, i.e. relapses during this period were not included in the calculation of baseline ARR. Between NTZ withdrawal and the initiation of fingolimod, 54 out of 252 patients (21.4%) had one relapse, and 21 out of 252 patients (8.3%) had at least two relapses. After treatment with NTZ, fingolimod was able to stabilize ARR, EDSS, and MRI activity in these patients, and controlled the disease, after 12, 24 and 36 months of treatment.

### Post-iDMT population

Our results indicated that Post-iDMT patients had a long time of disability, with high inflammatory activity (relapses and MRI activity), and approximately half of them had received at least one iDMT. Most of the Post-iDMT patients mainly switched to fingolimod due to the lack of effectiveness of the previous treatment. Fingolimod treatment reduced the number of relapses and MRI activity. Nevertheless, if comparing with naïve patients, fingolimod showed a higher efficacy in younger patients with less time of disability, reinforcing the importance of treating early and switching the treatment at the right time, and not too late. Most of the Post-iDMT patients receiving fingolimod also experienced an improvement in EDSS, or remained stable, during the whole study period. It is necessary to note that there are no data of patients receiving oral DMT (such as dimethyl fumarate or teriflunomide) because these drugs were not commercialized in Spain at the time of study initiation.

### Treatment persistence and safety profile in overall population

Fingolimod was a well-tolerated drug, with a high persistence after 12 months of treatment, in concordance with other studies.[6–8,11] In our study, AEs and lack of effectiveness were the main reasons of permanent discontinuation. Reported AEs, such as infections, liver enzymes increase, and lymphopenia < 200 cells/μl were consistent with the summary of product characteristics.

### Other studies with fingolimod in clinical practice

Several Spanish studies have demonstrated the efficacy of fingolimod in clinical practice, by reducing ARR and MRI lesion, and stabilizing EDSS.[22–24] All of these studies share the lower number of patients, compared with our present MS NEXT. Similarly, international studies (German, Italian, United Kingdom) have corroborated same beneficial results in real-world settings.[25,27,28]

### Limitations of the study

The methodology for evaluating some clinical outcomes (for instance, relapses or disability) may be somehow subjective; affecting comparisons with other studies.[29] Nevertheless, this limitation is intrinsically associated with studies in clinical practice. Moreover, potential differences with other international studies may derive from the fact that, in some countries, fingolimod is indicated as first-line agent, whereas in Spain is so as second one, after failure of a previous treatment. Another limitation in clinical practice is the MRI evaluation of stable patients that may not be performed annually, so the information regarding the percentage of patients free from disease should be cautiously interpreted. Besides limitations, MS NEXT is the study at a national level in real-world settings with fingolimod involving the largest cohort of patients.

## Conclusion

The subgroups of patients analysed showed differential demographic and clinical characteristics at baseline. The analysis of patients who received fingolimod in routine clinical practice confirmed an adequate profile of efficacy and safety, even for long-term treatment. These results corroborate those found in pivotal clinical trials. The present data also confirm the positive benefit/risk balance of using fingolimod in real-world clinical practice setting.

## Supporting information

S1 File List of MS NEXT study investigators. (DOCX)

S1 Fig Patients free from disease and patients free from clinical activity after 12 and 24 months of treatment with fingolimod. (TIF)

S1 Table Results of secondary endpoints for the total population and for patient subgroups regarding previous treatment. iDMTs, injectable disease-modifying therapies; NTZ, natalizumab; N.A., not available; EDSS, expanded disability status scale; CD, confirmed disability; MRI, magnetic resonance imaging.
(DOCX)
