# Supplementary material for: Spanish real-world experience with fingolimod in relapsing-remitting multiple sclerosis patients: MS NEXT study
Source: PLoS One. 2020 Apr 2;15(4):e0230846. doi: 10.1371/journal.pone.0230846 (PMC7117743; doi:10.1371/journal.pone.0230846)
Supplement: S1 File — (DOCX) [file pone.0230846.s001.docx]

The list of additional researchers participating in the MS NEXT study is as follows: Carmen Muñoz, MD, Complejo Universitario de Torrecárdenas, Almería (Spain); Fernando Sánchez, MD, Hospital Universitario Reina Sofía, Córdoba (Spain); Eduardo Agüera, MD, Hospital Universitario Reina Sofía, Córdoba (Spain); Carmen Arnal, MD, Hospital Virgen de las Nieves, Granada (Spain); Raúl Espinosa, MD, Hospital Puerta del Mar, Cádiz (Spain); Guillermo Navarro, MD, Hospital Universitario Virgen Macarena, Sevilla (Spain); Ricardo Fernández, MD, Hospital Universitario Virgen de Valme, Sevilla (Spain); Eduardo Durán, MD, Hospital Infanta Elena, Huelva (Spain); Jesús Martín, MD, Hospital Universitario Miguel Servet, Zaragoza (Spain); Carlos Tordesillas, MD, Hospital General San Jorge, Huesca (Spain); Ana María Latorre, MD, Hospital General San Jorge, Huesca (Spain); Agustín Oterino, MD, Hospital Universitario Marqués de Valdecilla, Santander (Spain); Vicente González, MD, Hospital Universitario Marqués de Valdecilla, Santander (Spain); Yasmina El Berdei, MD, Hospital Universitario Salamanca, Salamanca (Spain); Ana Belén Caminero, MD, Hospital Nuestra Señora de Sonsoles, Ávila (Spain); Luis Hernández, MD, Hospital de León, León (Spain); Laura Redondo, MD, Hospital de León, León (Spain); Yolanda Blanco, MD, Hospital Clínic Barcelona, Barcelona (Spain); Montse Artola, Hospital Clínic Barcelona, Barcelona (Spain); Antonio Escartín, MD, Hospital de la Santa Creu i Sant Pau, Barcelona (Spain); Nuria Vidal, Hospital de la Santa Creu i Sant Pau, Barcelona (Spain); Mariana López, Hospital de la Santa Creu i Sant Pau, Barcelona (Spain); Sebastián Figueroa, MD, Hospital de la Santa Creu i Sant Pau, Barcelona (Spain); Lluís Ramió i Torrentà, MD, Hospital Universitari Dr. Josep Trueta, Girona (Spain); Héctor Perkal, MD, Hospital Universitari Dr. Josep Trueta, Girona (Spain); Xavier Montalbán, MD, Multiple Sclerosis Centre of Catalonia (Cemcat), Hospital Universitari Vall d'Hebron, Barcelona (Spain); Ángela Vidal, MD, Multiple Sclerosis Centre of Catalonia (Cemcat), Hospital Universitari Vall d'Hebron, Barcelona (Spain); Joaquín Castillo, MD, Multiple Sclerosis Centre of Catalonia (Cemcat), Hospital Universitari Vall d'Hebron, Barcelona (Spain); Jaume Sastre, MD, Multiple Sclerosis Centre of Catalonia (Cemcat), Hospital Universitari Vall d'Hebron, Barcelona (Spain); Mar Tintoré, MD, Multiple Sclerosis Centre of Catalonia (Cemcat), Hospital Universitari Vall d'Hebron, Barcelona (Spain); Carlos Nos, MD, Multiple Sclerosis Centre of Catalonia (Cemcat), Hospital Universitari Vall d'Hebron, Barcelona (Spain); Breogán Rodríguez, MD, Multiple Sclerosis Centre of Catalonia (Cemcat), Hospital Universitari Vall d'Hebron, Barcelona (Spain); Luis Brieva Ruiz, MD, Hospital Universitari Arnau de Vilanova, Lleida (Spain); Anna Gil, Hospital Universitari Arnau de Vilanova, Lleida (Spain); Jorge Carlos Lecina, Hospital Universitari Arnau de Vilanova, Lleida (Spain); Desiree Muriana, Hospital de Mataró, Barcelona (Spain); Antonio Tomás Cano, MD, Hospital de Mataró, Barcelona (Spain); Sergio Martínez Yélamos, MD, Hospital Universitari Bellvitge, Barcelona (Spain); Elisabeth Matas, MD, Hospital Universitari Bellvitge, Barcelona (Spain); Isabel León, Hospital Universitari Bellvitge, Barcelona (Spain); Susana Pobla, Hospital Universitari Bellvitge, Barcelona (Spain); Laura Bau, MD, Hospital Universitari Bellvitge, Barcelona (Spain); Yolanda Aladro, MD, Hospital Universitario de Getafe, Madrid (Spain); Sara Moreno, MD, Hospital Universitario 12 de Octubre, Madrid (Spain); Ana Isabel Jimeno, MD, Hospital Universitario Puerta de Hierro, Madrid (Spain); Ruth García, Hospital Universitario Puerta de Hierro, Madrid (Spain); Irene del Pilar Moreno, MD, PhD, Hospital Universitario Puerta de Hierro, Madrid (Spain); Ricardo Ginestal, MD, PhD, Hospital Universitario Fundación Jiménez Díaz, Madrid (Spain); Eva María Ferrero, Hospital Clínico San Carlos, Madrid (Spain); Elena Guerra, MD, Hospital Clínico San Carlos, Madrid (Spain); José Carlos Álvarez Cermeño, MD, Hospital Ramon y Cajal, Madrid (Spain); Ambrosio Miralles, MD, Hospital Universitario Infanta Sofia, Madrid (Spain); Lluisa Rubio, MD, Hospital Universitario Príncipe de Asturias, Madrid (Spain); Lucía Ayuso, MD, Hospital Universitario Príncipe de Asturias, Madrid (Spain); Teresa Ayuso, MD, Complejo Hospitalario Navarra, Pamplona (Spain); María Otano, MD, Complejo Hospitalario Navarra, Pamplona (Spain); Lamberto Landete, MD, Hospital Universitario Doctor Peset, Valencia (Spain); Ángel Pérez, MD, Hospital General Universitario de Alicante (Spain); Juana Giménez, MD, Hospital General Universitario de Alicante, Alicante (Spain); Antonio Belenguer, MD, Hospital General Universitario de Castellón, Castellón (Spain); Javier Arnau, MD, Hospital General Universitario de Castellón, Castellón (Spain); María Carcelén, MD, Hospital General Universitario de Valencia, Valencia (Spain); Carlos Quintanilla, MD, Hospital General Universitario de Valencia, Valencia (Spain); Montserrat Gómez, MD, Hospital Universitario San Pedro de Alcántara, Cáceres (Spain); Pedro Enrique Jiménez, MD, Hospital Universitario San Pedro de Alcántara, Cáceres (Spain); Raúl Mauricio Romero, MD, Hospital Universitario San Pedro de Alcántara, Cáceres (Spain); José María Prieto, MD, Hospital Clínico Universitario Santiago, Santiago de la Compostela (Spain); Eva Costa, MD, Hospital Clínico Universitario Santiago, Santiago de la Compostela (Spain); Tania García, MD, Hospital Clínico Universitario Santiago, Santiago de la Compostela (Spain); Delicias Muñoz, MD, Hospital Xeral Cíes Vigo, Vigo (Spain); María del Campo, MD, Hospital Provincial de Pontevedra (CHOP), Pontevedra (Spain); Ana Rodríguez, MD, Hospital Provincial de Pontevedra (CHOP), Pontevedra (Spain); José Ramón Lorenzo, MD, Hospital Povisa, Vigo (Spain); José Antonio Cortés, MD, Hospital Lucus Augusti, Lugo (Spain); Leticia Álvarez, MD, Hospital Lucus Augusti, Lugo (Spain); Laura Ramos, MD, Hospital Lucus Augusti, Lugo (Spain); Miguel Ángel Llaneza, MD, Hospital Arquitecto Marcide, A Coruña (Spain); Carmen Calles, MD, Hospital Universitario Son Espases, Palma de Mallorca (Spain); Vanesa Núñez, MD, Hospital Universitario Son Espases, Palma de Mallorca (Spain); Margarita Massot, MD, Hospital Universitario Son Espases, Palma de Mallorca (Spain); Lucía Argandoña, MD, Hospital Mateu Orfila, Menorca (Spain); Gloria Llorens, MD, Hospital Mateu Orfila, Menorca (Spain); Jordi Ballabriga, MD, Hospital Son Llàtzer, Palma de Mallorca (Spain); Beatriz Romero, MD, Hospital Son Llàtzer, Palma de Mallorca (Spain); Miguel Ángel Hernández, MD, Hospital Universitario Nuestra Señora de Candelaria, Santa Cruz de Tenerife (Spain); Yolanda Marrero, MD, Hospital Universitario Nuestra Señora de Candelaria, Santa Cruz de Tenerife (Spain); Yessica Contreras, MD, Hospital Universitario Nuestra Señora de Candelaria, Santa Cruz de Tenerife (Spain); Javier Olascoaga, MD, Hospital Universitario Donostia, Donostia (Spain); Ioana Croitoru, MD, Hospital Universitario Donostia, Donostia (Spain); Francisco Julián-Villaverde, MD, Hospital Universitario Araba (HUA), Álava (Spain); Amaya Álvarez, MD, Hospital Universitario Araba (HUA), Álava (Spain); Javier Villafani, MD, Hospital Central Universitario de Asturias, Oviedo (Spain); Pedro Oliva, MD, Hospital Central Universitario de Asturias, Oviedo (Spain); Joaquín Peña, MD, Hospital de San Agustín, Avilés (Spain); Dionisio Fernández, MD, Hospital Universitario de Cabueñes, Gijón (Spain); Dulce María Solar, MD, Hospital Universitario de Cabueñes, Gijón (Spain); Roberto Suárez, MD, Hospital Universitario de Cabueñes, Gijón (Spain); Ester Carreón, MD, Hospital clínico Universitario Virgen de la Arrixaca, Murcia (Spain); José A. Pérez Vicente, MD, Hospital Santa Lucía, Murcia (Spain).
