# Supplementary figures and images for: Spanish real-world experience with fingolimod in relapsing-remitting multiple sclerosis patients: MS NEXT study
Source: PLoS One. 2020 Apr 2;15(4):e0230846. doi: 10.1371/journal.pone.0230846 (PMC7117743; doi:10.1371/journal.pone.0230846)

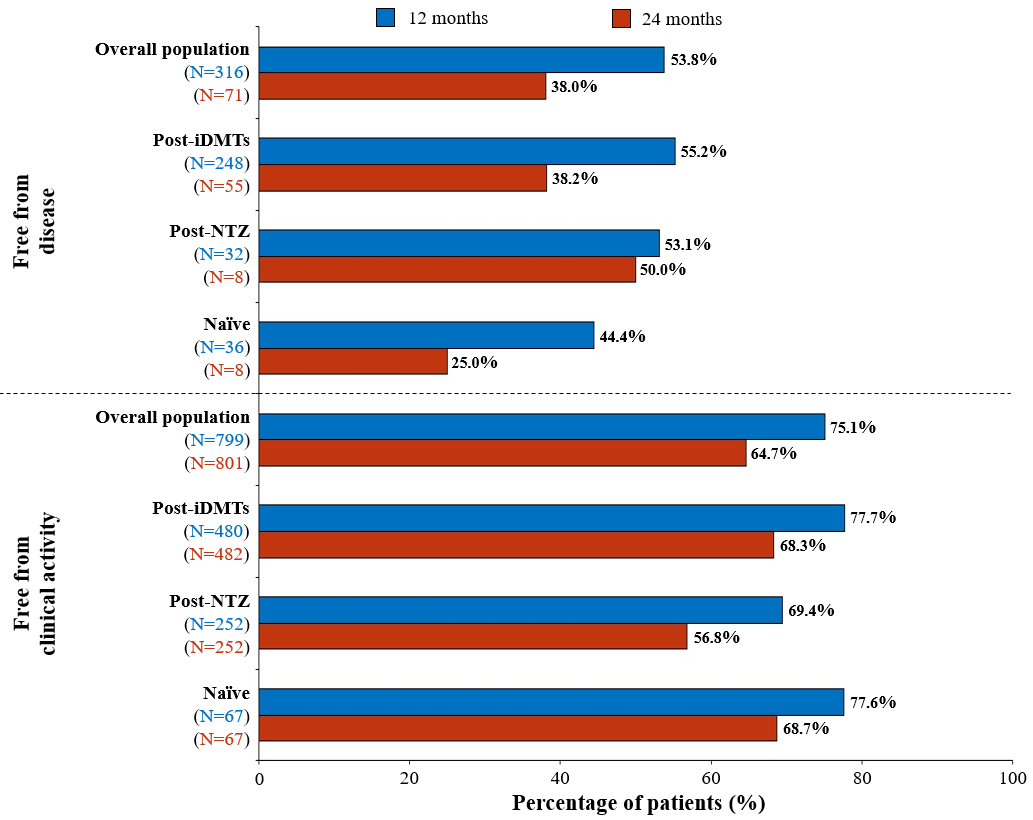

Supplement: S1 Fig — (TIF) [file pone.0230846.s002.tif]
