# Supplementary material for: Spanish real-world experience with fingolimod in relapsing-remitting multiple sclerosis patients: MS NEXT study
Source: PLoS One. 2020 Apr 2;15(4):e0230846. doi: 10.1371/journal.pone.0230846 (PMC7117743; doi:10.1371/journal.pone.0230846)
Supplement: S1 Table — iDMTs, injectable disease-modifying therapies; NTZ, natalizumab; N.A., not available; EDSS, expanded disability status scale; CD, confirmed disability; MRI, magnetic resonance imaging. (DOCX) [file pone.0230846.s003.docx]

|  | **Post-iDMTs** | **Post-NTZ** | **Naïve** | **TOTAL** | **P-value (inter-group)** |
| --- | --- | --- | --- | --- | --- |
| **Time to first relapse**, mean months (SD) | 27.4 (0.6) | 22.0 (0.7) | 25.5 (1.3) | 26.6 (0.5) | 0.024 |
| **EDSS**, mean (95% CI) |  |  |  |  |  |
| Previous year | 2.9 (2.8 to 3.1) | 3.5 (3.3 to 3.7) | 2.7 (2.2 to 3.1) | 3.1 (3.0 to 3.2) | <0.001 |
| n available | 485 | 252 | 67 | 804 |  |
| After 12 months | 2.8 (2.7 to 2.9) | 3.7 (3.4 to 3.9) | 2.4 (2.0 to 2.9) | 3.0 (2.9 to 3.2) | <0.001 |
| n available | 485 | 252 | 67 | 804 |  |
| After 24 months | 2.9 (2.7 to 3.2) | 3.6 (3.3 to 3.9) | 2.6 (1.9 to 3.4) | 3.1 (3.0 to 3.3) | 0.001 |
| n available | 230 | 139 | 33 | 402 |  |
| After 36 months | 3.0 (2.6 to 3.5) | 3.8 (3.2 to 4.3) | 1.6 (0.5 to 2.7) | 3.2 (2.9 to 3.5) | 0.004 |
| n available | 69 | 50 | 9 | 128 |  |
| **3-month CD,** n (%) | 36 (7.4) | 39 (15.5) | 7 (10.5) | 82 (10.2) | 0.008 |
| Patients free from CDP, n (%) | 442 (91.1) | 212 (84.1) | 59 (88.1) | 713 (88.7) |  |
| After 12 months | 469 (96.6) | 235 (93.3) | 63 (94.0) | 767 (95.4) |  |
| After 24 months | 481 (99.4) | 245 (97.2) | 66 (98.5) | 792 (98.5) |  |
| After 36 months | 484 (99.8) | 251 (99.6) | 66 (98.5) | 801 (99.6) |  |
| **Number of gadolinium-enhancing T1 lesions,** mean (95% CI) |  |  |  |  |  |
| Previous year | 1.3 (1.0 to 1.6) | 0.3 (-0.1 to 0.6) | 1.7 (0.8 to 2.5) | 1.2 (1.0 to 1.4) | <0.001 |
| n available | 307 | 57 | 40 | 404 |  |
| After 12 months | 0.3 (0.2 to 0.5) | 0.4 (0.1 to 0.8) | 0.6 (0.3 to 0.9) | 0.4 (0.3 to 0.5) | 0.027 |
| n available | 252 | 34 | 36 | 322 |  |
| After 24 months | 0.1 (0.0 to 0.2) | 0.1 (-0.1 to 0.2) | 0.3 (-0.2 to 0.7) | 0.1 (0.0 to 0.2) | 0.465 |
| n available | 89 | 15 | 11 | 115 |  |
| After 36 months | 0.4 (-0.4 to 1.1) | 0.3 (-0.6 to 1.1) | 0.0 (0.0) | 0.3 (-0.2 to 0.8) | 0.418 |
| n available | 17 | 4 | 4 | 25 |  |
| **T2 lesions,** n (%) |  |  |  |  |  |
| Previous year |  |  |  |  |  |
| n available | 298 | 54 | 40 | 392 |  |
| <9 | 24 (8.1) | 4 (7.4) | 7 (17.5) | 35 (8.9) | 0.160 |
| 9-20 | 113 (37.9) | 28 (51.9) | 13 (32.5) | 154 (39.3) | 0.101 |
| >20 | 161 (54.0) | 22 (40.7) | 20 (50.0) | 203 (51.8) | 0.193 |
| After 12 months |  |  |  |  |  |
| n available | 263 | 36 | 36 | 322 |  |
| New/enlarged T2 lesions | 64 (24.3) | 6 (16.7) | 14 (38.9) | 84 (25.1) | 0.116 |
| <9 | 13 (20.3) | 0 (0.0) | 3 (21.4) | 16 (19.1) |  |
| 9-20 | 23 (35.9) | 0 (0.0) | 3 (21.4) | 26 (31.0) |  |
| >20 | 27 (42.2) | 6 (100.0) | 6 (42.9) | 39 (46.4) |  |
| After 24 months, n (%) |  |  |  |  |  |
| n available | 96 | 15 | 12 | 123 |  |
| New/enlarged T2 lesions | 30 (31.3) | 3 (20.0) | 3 (25.0) | 36 (29.3) | 0.702 |
| <9 | 7 (23.3) | 0 (0.0) | 0 (0.0) | 7 (19.4) |  |
| 9-20 | 9 (30.0) | 3 (100.0) | 2 (66.7) | 14 (38.9) |  |
| >20 | 14 (46.7) | 0 (0.0) | 1 (33.3) | 15 (41.7) |  |
| After 36 months |  |  |  |  |  |
| n available | 19 | 4 | 4 | 27 |  |
| New/enlarged T2 lesions | 3 (15.8) | 0 (0.0) | 3 (75.0) | 6 (22.2) | 0.444 |
